# The Utility of PET/CT Metabolic Parameters Measured Based on Fixed Percentage Threshold of SUVmax and Adaptive Iterative Algorithm in the New Revised FIGO Staging System for Stage III Cervical Cancer

**DOI:** 10.3389/fmed.2021.680072

**Published:** 2021-07-29

**Authors:** Yun Zhang, Yuxiao Hu, Shuang Zhao, Can Cui

**Affiliations:** Department of PET/CT Center, Jiangsu Cancer Hospital and Jiangsu Institute of Cancer Research and The Affiliated Cancer Hospital of Nanjing Medical University, Nanjing, China

**Keywords:** cervical cancer, 18F-FDG-PET/CT metabolic parameters, revised FIGO staging system, fixed percentage threshold of SUVmax, AT-AIA

## Abstract

**Objectives:** The main aim of this study was to evaluate the differences in metabolic parameters of positron emission tomography with 2-deoxy-2-[fluorine-18] fluoro-D-glucose integrated with computed tomography (^18^F-FDG PET/CT) measured based on fixed percentage threshold of maximum standard uptake value (SUVmax) and adaptive iterative algorithm (AT-AIA) in patients with cervical cancer. Metabolic parameters in stage III patients subdivided into five groups according to FIGO and T staging (IIIB-T3B, IIIC1-T2B, IIIC1-T3B, IIIC2-T2B, IIIC2-T3B) were compared.

**Methods:** In total, 142 patients with squamous cell cervical cancer subjected to ^18^F-FDG-PET/CT before treatment were retrospectively reviewed. SUVmax, mean standard uptake value (SUVmean), maximum glucose homogenization (GNmax), mean glucose homogenization (GNmean), metabolic tumor volume (MTV), total lesion glycolysis (TLG), and glucose homogenization total lesion glycolysis (GNTLG) values measured based on the above two measurement methods of all 142 patients (IIB-IVB) and 102 patients in the above five groups were compared.

**Results:** MTV measured based on fixed percentage threshold of SUVmax was lower than that based on AT-AIA (*p* < 0.05). MTV_40%_, MTV_0.5_, TLG_0.5_, GNTLG_40%_, and GNTLG_0.5_ values were significantly different among the five groups (*p* < 0.05) while the rest parameters were comparable (*p* > 0.05). All metabolic parameters of group IIIB-T3B were comparable to those of the other four groups. MTV_40%_, MTV_0.5_, GNTLG_40%_, and GNTLG_0.5_ in group IIIC1-T2B relative to IIIC1-T3B and those of group IIIC2-T2B relative to group IIIC2-T3B were significantly different. All metabolic parameters of group IIIC1-T2B relative to IIIC2-T2B and those of group IIIC1-T3B relative to group IIIC2-T3B were not significantly different.

**Conclusion:** Metabolic parameters obtained with the two measurement methods showed a number of differences. Selection of appropriate methods for measurement of ^18^F-FDG-PET/CT metabolic parameters is important to facilitate advances in laboratory research and clinical applications. When stage III patients had the same T stage, their metabolic parameters of local tumor were not significantly different, regardless of the presence or absence of lymph node metastasis, location of metastatic lymph nodes in the pelvic cavity or para-abdominal aorta. These results support the utility of the revised FIGO system for stage III cervical cancer, although our T-staging of stage III disease is incomplete.

## Introduction

Globally, cervical cancer is one of the most common cancer types in females, ranking fourth after breast, colorectal, and lung cancer in terms of morbidity and mortality (1). Cervical cancer has been relatively well-controlled for several decades in high-income countries owing to efficient screening initiatives and cancer treatment services but remains the most common cause of cancer-related mortality in 42 countries, the majority of which are low income and lower-middle income countries (LMIC) (2), such as South Africa (SA), India, China, and Brazil.

Gynecologic cancers are staged according to the International Federation of Gynecology and Obstetrics (FIGO) system (3). Although a parallel TNM system has been described by the American Joint Committee on Cancer, the FIGO system continues to be predominantly used worldwide in clinical practice and for cancer database reporting (4). In 2018, FIGO revised the staging system for cervical cancer based on recent developments in imaging and increased use of minimally invasive surgery, which has changed the paradigm for management of this patient group. One of the modifications in the revised FIGO system is that nodal status is incorporated into the criteria for stage III disease. Consequently, cases of lymph node metastasis are designated stage IIIC disease, specifically, stage IIIC1 for pelvic lymph node metastasis only, and stage IIIC2 for para-aortic lymph node metastasis (3).

Imaging plays a central role in the 2018 FIGO staging system for uterine cervical cancer. ^18^F-FDG-PET/CT has significant advantages in detecting lymph node metastases and distant metastases (5). For cervical cancer, ^18^F-FDG-PET/CT metabolic parameters of primary tumors and lymph nodes, such as SUVmax, MTV, and TLG, have considerable value (6–14). Yilmaz et al. (6) identified pretreatment primary tumor SUVmax, TLG, pelvic lymph node SUVmax, and pretreatment para-aortic lymph node SUVmax as significant prognostic factors for disease-free survival (DFS) with different cut-off values. The group of Lima showed that pretreatment MTV and TLG values and nodal involvement were effective predictors of response to therapy in a cohort with locally advanced squamous cell cervical cancer (LACC) patients treated with computer-controlled radiation therapy (CCRT). MTV was identified as the best predictor of response (11). Xu et al. (14) reported a combination of tumor TLG, Dmin [obtained by the diffusion-related coefficient (D) map of MRI] and PET for lymph node diagnosis as a powerful prognostic factor for cervical cancer. TLG showed the best predictive performance in patients with PET-negative lymph nodes.

The most commonly used metabolic parameter to quantify ^18^F-FDG uptake on PET is the SUVmax. The SUVmax was widely accepted and routinely clinical used owing to the ease of use and an excellent inter-observer reproducibility in association with promising results for SUVmax as a prognostic factor (15, 16). However, the use of SUVmax has many disadvantages, especially the variability caused by the high statistical noise associated with a single voxel analysis (17). TLG was proposed as an alternative quantitative metric in 1999, which take the SUV and the tracer uptake of the entire lesion into account. TLG is defined as the MTV multiplied with the SUVmean. The MTV is determined as the total number of voxels within a volume of interest (VOI) that have uptake above a predetermined SUV threshold (18). Various automated methods are currently used to segment regions of interest in PET/CT scans, such as fixed SUV threshold (e.g., SUV2.5), fixed percentage threshold of SUVmax (e.g., T42%), and gradient-based threshold (adaptive iterative algorithm, AT-AIA) (19). At present, the fixed percentage of the SUVmax threshold algorithm is commonly used, especially for target delineation of cervical cancer, lung cancer, and head and neck cancers (6, 11, 20–22). In 2006, Sebastian et al. (23) published the iterative adaptive segmentation algorithm. The adaptive iterative algorithm has an advantage over fixed threshold methods in accurate delineation of the target volume according to the individual metabolic activity. This method is usually based on the SUVmax uptake within the volume and the threshold defined according to the background uptake within the adjacent normal tissue using a mathematical algorithm.

In view of the wide application of the ^18^F-FDG-PET/CT metabolic parameters in cervical cancer and the MTV and TLG are greatly affected by the different measurement methods, one of the major objectives of the current investigation was to compare the ^18^F-FDG-PET/CT metabolic parameters obtained using the fixed percentage threshold of SUVmax and AT-AIA in patients with LACC.

According to the new revised FIGO staging system, Stage IIIC disease is directly correlated with pelvic and para-aortic metastatic lymph nodes regardless of the T stage. We additionally focused on differences in ^18^F-FDG-PET/CT metabolic parameters of local tumors with different T-stages in patient groups of stage III cervical cancer.

## Patients and Methods

### Patients

The clinical records of all patients referred to our center for cervical cancer from May 2016 to July 2020 were analyzed. In total, 142 patients with squamous cell cervical cancer confirmed via biopsy were included.

All patients underwent routine clinical staging, including physical and gynecological examinations, complete blood count, biochemical tests, and radiological imaging of the pelvis and abdomen with enhanced MRI or enhanced CT. Patients with histologically confirmed cervical cancer (FIGO stage IIB–IVB) underwent a ^18^F-FDG-PET/CT examination before treatment.

### PET/CT Imaging

All patients were imaged using an integrated PET/CT system (Discovery 710, GE Medical Systems, Waukesha, Wisconsin, USA). Patients fasted for at least 6 h before intravenous administration of 0.1–0.2 mCi/kg ^18^F-FDG. Blood glucose concentrations were measured before the injection of radiopharmaceuticals to ensure a threshold <11 mmol/L. Patients were allowed to rest during distribution of the radiotracer in a comfortable, quiet room, and hydrated orally with 1,000 ml water. Patients were instructed to empty their bladder immediately before the scan. Combined image acquisition began about 50–70 min after ^18^F-FDG injection. From the vertex to mid-thigh, CT was performed using the following parameters: 140 kV, Auto mA (noise index, 28.5), 0.8 s rotation time, and 3.75 mm slice thickness. A PET scan was performed with the same parameters. The emission scan time was 2 min/bed position and the scanning range covered 6–7 bed positions. PET image datasets were reconstructed iteratively using the ordered-subsets expectation maximization algorithm with CT-based attenuation correction. The following parameters were used: sharp IR algorithm with the VUE Point FX (fully 3D iterative reconstruction), 192×192 matrix, 24 subset/2 iteration, and 6.4 post-filter. Trans axial, sagittal, coronal, and fused images were analyzed on an Advanced Workstation AW 4.6 (GE Healthcare Bio-Sciences, NJ, USA).

### PET/CT Image Analysis

Qualitative and quantitative (or semi-quantitative) image analyses were conducted by an experienced nuclear medicine physician with significant experience in ^18^F-FDG-PET/CT scan analysis (average 150 reads/month individually). A VOI was placed around the primary tumor in such a way that the entire tumor activity was enclosed and regions of physiologically increased activity avoided. VOI placement was performed according to a previously published protocol (24). Within the selected VOI, SUVmax, SUVmean, GNmax, GNmean, MTV, TLG, and GNTLG [SUV is a measurement of the uptake in a tumor normalized on the basis of a distribution volume. GN is defined as SUV with plasma glucose correction. SUVmax and GNmax are the maximum SUV and GN. SUVmean and GNmean are the mean SUV and GN (15). The MTV is determined as the total number of voxels within a volume of interest that have uptake above a predetermined SUV threshold. TLG was defined as the MTV multiplied with the SUVmean (18). GNTLG was defined as the MTV multiplied with the GNmean.] were measured based on fixed percentage threshold (40% SUVmax) [All voxels with SUVs above or equal to 40% of the SUVmax were delineated inside the selected VOI (6, 8).] and AT-AIA [The VOI was segmented automatically using an iterative adaptive algorithm to detect the threshold level that separated the target volume from the background tissue by weighting the SUVmax and the SUVmean within the target volume with a weighting factor “w” (0 ≤ w ≤ 1)]. This weighting factor was automatically set at 0.5 (19, 21, 22, 25).

### Statistical Analysis

Comparisons between the two groups were performed with the independent samples *t*-test or Mann-Whitney U-test depending on the homogeneity of variance. Multi-group comparisons were conducted with ANOVA. All hypotheses were two-tailed and *P* < 0.05 considered statistically significant. Statistical Package for Social Sciences (SPSS, version 22.0, IBM Corp, Armonk, NY, USA) was applied for data analysis.

## Results

### Clinical Features and PET Metabolic Parameters of the Two Groups Measured Using Different Methods

^18^F-FDG-PET/CT metabolic parameters obtained with the two methods were compared in 142 patients with squamous cell cervical cancer. The clinical characteristics of participants are listed in Table 1. Mean age of patients was 53.39 ± 9.61 years. The most common FIGO stage was IIIC1 (*n* = 53, 37.32%) followed by IIIC2 (*n* = 32, 22.50%), IVB (*n* = 27, 19.01%), IIIB (*n* = 19, 13.38%), IIB (*n* = 9, 6.34%), IIIA (*n* = 1, 0.70%), and IVA (*n* = 1, 0.70%).

**Table 1:** Clinical characteristics of patients with squamous cell cervical cancer.

	**IIB**	**IIIA**	**IIIB**	**IIIC1**	**IIIC2**	**IVa**	**IVb**
Number, %	9 (6.34%)	1 (0.70%)	19 (13.38%)	53 (37.32%)	32 (22.54%)	1 (0.70%)	27 (19.01%)
Age (year)	55.89 ± 5.18	66	58.84 ± 11.49	51.77 ± 8.72	50.81 ± 10.11	49	54.67 ± 9.02
Stature (cm)	158.33 ± 7.04	152	157.95 ± 4.45	160.85 ± 5.18	159.38 ± 5.11	155	158.04 ± 5.41
Weight (kg)	60.56 ± 6.41	71	58.79 ± 8.44	60.11 ± 8.54	60.02 ± 9.31	49	59.06 ± 9.86
^18^F-FDG dose (mCi/kg)	0.16 ± 0.02	0.14	0.18 ± 0.04	0.17 ± 0.03	0.17 ± 0.04	0.22	0.17 ± 0.03
Blood glucose (mmol/L)	5.54 ± 1.34	5.70	5.81 ± 1.51	5.62 ± 1.10	5.57 ± 0.63	6.10	5.61 ± 0.71

**Figure 1:**
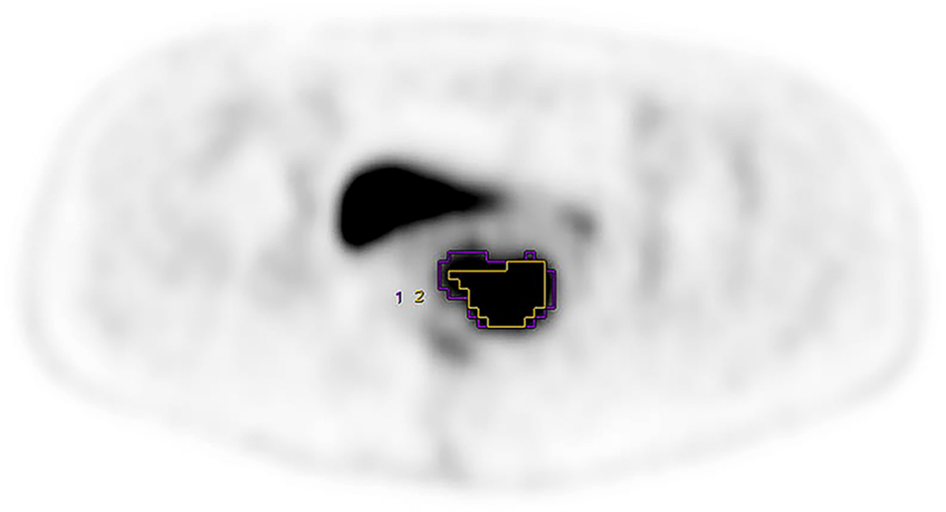
FIGO stage was IIIB, purple VOI and yellow VOI were measured based on the AT-AIA(*w* = 0.5) and fixed percentage threshold (40% SUVmax), respectively. MTV_0.5_ = 32.16, MTV_40%_ = 17.30.

PET parameters of the two groups are listed in Table 2. We observed no significant differences in SUVmean, GNmean, TLG, and GNTLG values between the groups. MTV measured based on fixed percentage threshold (40% SUVmax) was lower than that based on AT-AIA (*w* = 0.5, Figure 1).

**Table 2:** PET parameters of groups measured using the two different methods.

	**Fixed percentage threshold**	**AT-AIA**	***P***
SUVmean	9.32 ± 3.59	8.60 ± 3.21	0.074 (*t* = −1.79)
GNmean	9.31 ± 3.44	8.55 ± 3.14	0.051 (*t* = −1.96)
MTV (cm^3^)	28.64 (15.40–50.71)	35.84 (21.17–60.91)	0.019 (*Z* = −2.34)
TLG	276.40 (121.24–500.10)	315.15 (145.55–535.43)	0.22 (*Z* = −1.22)
GNTLG	277.91 (124.04–503.02)	319.27 (151.13–559.39)	0.24 (*Z* = −1.18)

*SUVmax = 15.48 ± 5.86*.

### Patient Characteristics and PET Parameters of IIIB-IIIC2 Groups

To establish whether metabolic ^18^F-FDG-PET/CT parameters of local tumors at various T-stages differ among patients with stage III cervical cancer, 102 patients from groups IIIB-IIIC2 were analyzed. SUVmean, GNmean, MTV, TLG, GNTLG measured based on fixed percentage threshold (40% SUVmax) and AT-AIA (w = 0.5) were labeled SUVmean_40%_, GNmean_40%_, MTV_40%_, TLG_40%_, GNTLG_40%_, and SUVmean_0.5_, GNmean_0.5_, MTV_0.5_, TLG_0.5_, and GNTLG_0.5_, respectively. Patients were subdivided into five groups according to FIGO system and T staging of the TNM system: IIIB-T3B, IIIC1-T2B, IIIC1-T3B, IIIC2-T2B, and IIIC2-T3B. Patient characteristics and PET parameters of IIIB-IIIC2 groups are listed in Table 3.

**Table 3:** Patient characteristics and PET parameters (mean ± *SD*) of groups IIIB-IIIC2.

	**IIIB-T3B**	**IIIC1-T2B**	**IIIC1-T3B**	**IIIC2-T2B**	**IIIC2-T3B**	***P***
Number, %	19 (18.63%)	26 (25.49%)	25 (24.51%)	15 (14.71%)	17 (16.67%)	
Age (year)	56.58 ± 13.18	52.62 ± 9.88	52.08 ± 6.51	51.00 ± 12.85	50.65 ± 7.32	0.401 (*F* = 1.020)
Stature (cm)	158.63 ± 4.22	160.50 ± 5.60	161.48 ± 4.58	159.40 ± 5.11	159.35 ± 5.27	0.363 (*F* = 1.096)
Weight (kg)	60.53 ± 7.49	59.69 ± 7.23	60.20 ± 10.04	59.13 ± 10.85	60.79 ± 7.96	0.983 (*F* = 0.099)
^18^F-FDG dose (mCi/kg)	0.17 ± 0.04	0.17 ± 0.03	0.17 ± 0.04	0.16 ± 0.03	0.17 ± 0.04	0.978 (*F* = 0.112)
Blood glucose (mmol/L)	5.75 ± 1.53	5.51 ± 1.15	5.79 ± 1.06	5.51 ± 0.65	5.62 ± 0.63	0.869 (*F* = 0.313)
SUVmax	14.56 ± 5.89	16.57 ± 6.64	17.56 ± 7.51	13.29 ± 5.18	15.29 ± 4.34	0.233 (*F* = 1.420)
GNmax	14.48 ± 4.04	16.28 ± 6.41	17.76 ± 7.27	13.04 ± 4.60	15.33 ± 4.16	0.137 (*F* = 1.791)
SUVmean_40%_	8.70 ± 3.50	9.94 ± 4.15	10.53 ± 4.58	8.19 ± 3.46	9.09 ± 2.66	0.308 (*F* = 1.218)
SUVmean_0.5_	8.01 ± 3.20	9.13 ± 3.57	9.39 ± 4.03	7.94 ± 3.32	8.51 ± 2.49	0.570 (*F* = 0.736)
GNmean_40%_	8.64 ± 3.23	9.89 ± 3.87	10.65 ± 4.48	8.02 ± 3.06	9.11 ± 2.56	0.172 (*F* = 1.633)
GNmean_0.5_	7.80 ± 3.01	8.94 ± 3.39	9.51 ± 3.98	7.78 ± 2.92	8.37 ± 2.84	0.441 (*F* = 0.945)
MTV_40%_(cm^3^)	42.02 ± 37.29	30.56 ± 14.41^bd^	46.29 ± 28.50^ac^	27.09 ± 21.51^bd^	50.07 ± 32.21^ac^	0.046 (*F* = 2.516)
MTV_0.5_ (cm^3^)	47.64 ± 36.49	39.75 ± 19.23^b^	59.25 ± 36.78^ac^	29.08 ± 21.67^bd^	58.05 ± 31.85^c^	0.014 (*F* = 3.286)
TLG_40%_	359.67 ± 355.08	315.89 ± 200.96	448.19 ± 270.73	226.46 ± 174.11	474.71 ± 344.02	0.056 (*F* = 2.395)
TLG_0.5_	384.45 ± 353.89	368.74 ± 233.30	516.41 ± 311.47^c^	237.74 ± 185.41^b^^d^	519.57 ± 354.48^c^	0.028 (*F* = 2.839)
GNTLG_40%_	356.30 ± 243.14	309.05 ± 193.24^b^	466.29 ± 305.99^a^^c^	220.03 ± 164.41^b^^d^	475.66 ± 346.81^c^	0.033 (*F* = 2.740)
GNTLG_0.5_	380.26 ± 340.60	361.77 ± 225.32^b^	530.76 ± 346.67^a^^c^	231.08 ± 175.02^b^^d^	515.06 ± 358.32^c^	0.020 (*F* = 3.082)

a *significantly different from IIIC1-T2B*.

b *significantly different from IIIC1-T3B*.

c *significantly different from IIIC2-T2B*.

d *significantly different from IIIC2-T3B*.

*no significant difference between group IIIB-T3B and other groups*.

We observed no significant differences in age, stature, weight, ^18^F-FDG dose, blood glucose, SUVmax, SUVmean_40%_, SUVmean_0.5_, GNmax, GNmean_40%_, GNmean_0.5_, and TLG_40%_ values among the groups, with (F, P) of (1.020, 0.401), (1.096, 0.363), (0.099, 0.983), (0.112, 0.978), (0.313, 0.869), (1.420, 0.233), (1.218, 0.308), (0.736, 0.570), (1.791, 0.137), (1.633, 0.172), (0.945, 0.441), and (2.395, 0.056), respectively. However, MTV_40%_, MTV_0.5_, TLG_0.5_, GNTLG_40%_, and GNTLG_0.5_ were significantly different among the five groups, with (F, P) of (2.516, 0.046), (3.286, 0.014), (2.839, 0.028), (2.740, 0.033), and (3.082, 0.020), respectively.

**Figure 2:**
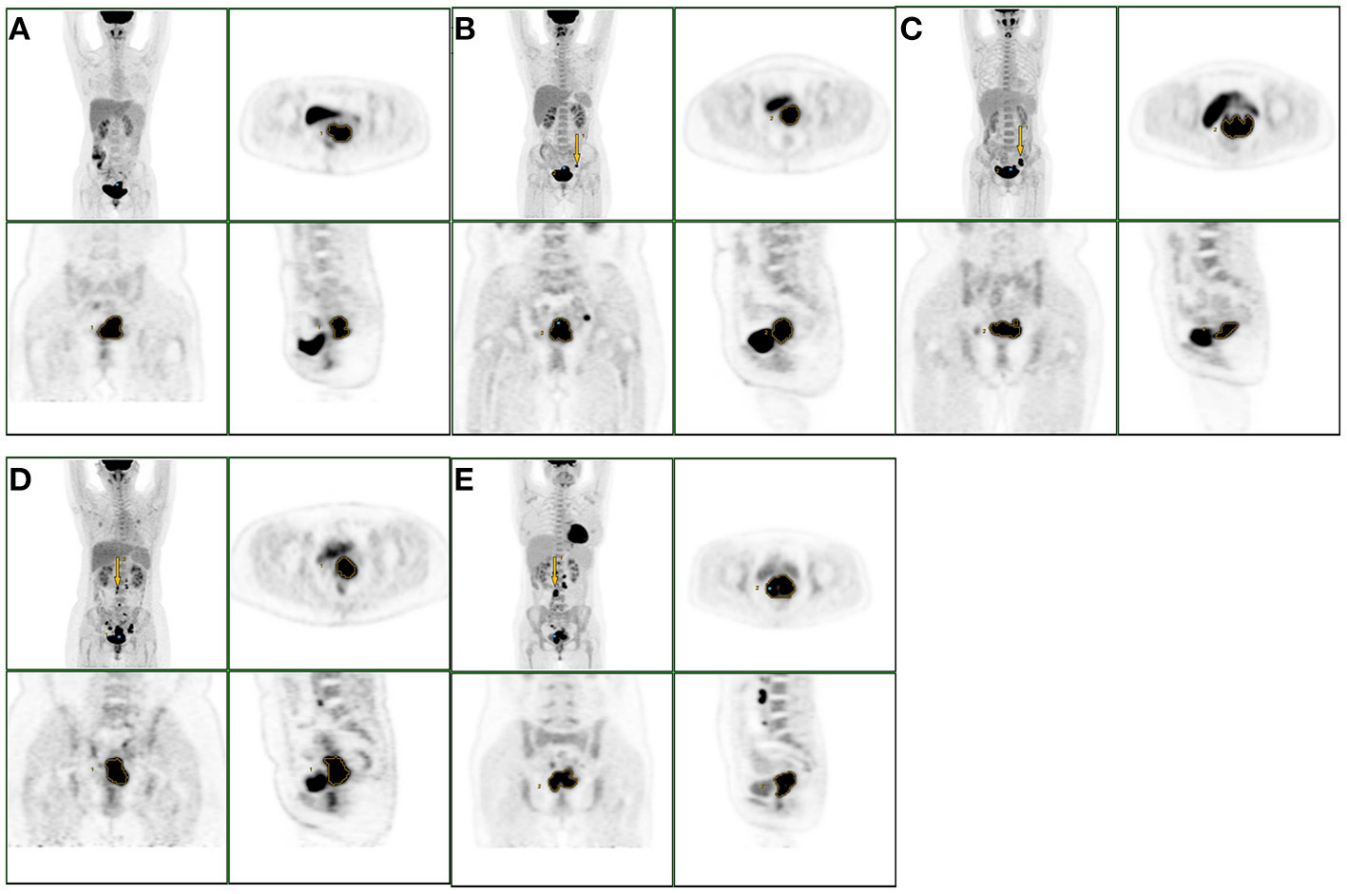
The group of A, B, C, D, E were IIIB-T3B, IIIC1-T2B, IIIC1-T3B, IIIC2-T2B, IIIC2-T3B. The yellow arrows indicated the metastatic lymph nodes. When stage III patients had the same T stage, their metabolic parameters of local tumor were not significantly different, regardless of the presence or absence of lymph node metastasis, location of metastatic lymph nodes in the pelvic cavity or para-abdominal aorta.

All metabolic parameters of group IIIB-T3B were comparable to those of the other four groups. Metabolic parameters of group IIIC1-T2B relative to IIIC2-T2B and those of IIIC1-T3B relative to IIIC2-T3B were not significantly different. MTV_40%_, MTV_0.5_, GNTLG_40%_, and GNTLG_0.5_ values of group IIIC1-T2B were lower than those of IIIC1-T3B while the TLG_0.5_ were comparable. MTV_40%_, MTV_0.5_, TLG_0.5_, GNTLG_40%_, and GNTLG_0.5_ values of group IIIC2-T2B were lower than those of group IIIC2-T3B. All metabolic parameters of group IIIC1-T2B or IIIC2-T2B were comparable to those of the group IIIB-T3B. MTV_40%_ of group IIIC1-T2B were lower than those of group IIIC2-T3B while the other metabolic parameters were comparable. MTV_40%_, MTV_0.5_, TLG_0.5_, GNTLG_40%_, and GNTLG_0.5_ values of group IIIC2-T2B were lower than those of group IIIC1-T3B (Figure 2).

## Discussion

MTV measured based on fixed percentage threshold (40% SUVmax) was lower than that based on the AT-AIA (*w* = 0.5). Weina Xu et al. (25) compared the accuracy of MTV by the iterative adaptive algorithm (MTViterative adaptive) with that of the fixed percentage SUVmax threshold method using gross tumor volume (GTV) as the gold standard and investigated the correlation between them. Significant differences were observed among the fixed percentage method and the optimal threshold percentage was inversely correlated with SUV max. MTViterative adaptive is independent of SUVmax, more accurate, and correlated with GTV in patients with early-stage cervical cancer (stage Ia–IIb). They speculated that iterative adaptive algorithm segmentation may be more suitable than the fixed percentage threshold method to estimate the tumor volume of cervical primary squamous cell carcinoma. The group of Xiao-Yi Wang investigated the suitable segmentation method in small, low uptake, and heterogeneous nodules of stage I lung adenocarcinoma and found that AT-AIA had the highest accuracy in large, high uptake, and solid nodules (19). This finding may be explained by phantom results showing that a fixed threshold can substantially underestimate MTV for lesions with high ^18^F-FDG uptake (26). Recent studies have reported limitations of this threshold method in measurement of lesion activity and volume. MTV and TLG values obtained based on a fixed threshold using SUVmax (40%) can lead to underestimation of lesional uptake with high activity and overestimation of lesions with SUVmax close to the background level (26). If the radiotherapy regimen is based on the measurement range of MTV with a fixed threshold using SUVmax, active tumor lesions are likely to be overestimated or underestimated.

On the other hand, the TLG and GNTLG values in our study were not significantly different between the two groups, the two measurement methods had no effect on TLG and GNTLG, which differs from the previous studies (26). This conclusion needs further study.

MTV measurements using different methods have been reported. Some studies have measured MTV in cervical cancer based on fixed percentage threshold (40% SUVmax) (6, 11, 21), while others have shown that MTV and TLG calculated using a threshold of 55% SUVmax and 32% SUVmax from pre- and per-treatment PET scans, respectively, could be effectively used to predict patient outcomes after CCRT for LACC (27). Burger and co-workers reported that PET volume metrics based on fixed SUVmax threshold (42%) led to significant bias and were not correlated with response to chemotherapy assessed via histopathologic examination, while PET volume metrics based on background-adapted measurements were correlated with tumor regression in non-small cell lung carcinoma (22). Other researchers used a fixed SUV threshold, most commonly 2.5 (27–30), with the obvious limitations of an arbitrary cutoff. However, lesions with low activity may have been consequently underestimated. In our study, MTV was also assessed with the fixed SUV threshold of 2.5 but bladder and rectum were incorporated into the VOI, which could potentially increase the value. Therefore we only compared the differences in ^18^F-FDG-PET/CT metabolic parameters measured based on fixed percentage threshold of 40% SUVmax and AT-AIA (*w* = 0.5). Metabolic parameters obtained using the two measurement methods showed some variations.

Currently, validation of methods for tumor quantification against published MTV and TLG is a challenge due to the lack of a true gold standard. There are some differences in MTV and TLG obtained by different measurement methods. Therefore, selection of the right measurement method is crucial to facilitate advances in research or clinical application.

According to the new revised FIGO staging system, Stage IIIC disease is directly related to pelvic and para-aortic metastatic lymph nodes regardless of the T stage. This new staging system clearly reflects the importance of lymph node metastasis as a major prognostic factor in cervical cancer. Matsuo and co-workers reported that stage IIIC1 is independently associated with improved cause-specific survival compared to stage IIIA or stage IIIB disease (5-year survival rates of 46.0% for stage IIIA, 42.6% for stage IIIB, and 62.1% for stage IIIC1 disease). Survival of patients with stage IIIC1 disease varied in a manner dependent on T-stage (5-year cause-specific survival rates: 74.8% for T1 stage, 58.7% for T2 stage, and 39.3% for T3 stage), indicating that local tumor factors in addition to nodal status are important determinants of survival (31). Many studies showed MTV and TLG of primary tumor were predictors of response to therapy and prognosis (6, 9, 11, 27). In other studies, TLG of the primary tumor has been used to construct a predictive model of lymph node metastasis (14, 21). Their findings suggest that the internal metabolism of the primary tumor may exert an effect on lymph node metastasis.

Inspired by the above studies, we attempted to investigate the differences of metabolic parameters of primary tumor in stage III, considering the different T staging and lymph node metastasis. Since there was only one patient in the stage IIIA in our study, we included the patients in stage IIIB, IIIC1, and IIIC2 in our study, and subdivided the patients into five groups according to the new revised FIGO staging system and T staging of the TNM system: IIIB-T3B, IIIC1-T2B, IIIC1-T3B, IIIC2-T2B, and IIIC2-T3B. According to our results, when the stage III patients have the same T stage, their metabolic parameters of local tumor were not significantly different. The differences between them were the presence or absence of lymph node metastasis, location of metastatic lymph nodes in the pelvic cavity or para-abdominal aorta. In stage IIIC1 or stage IIIC2, all patients with lymph node metastasis, the lower the T stage, the lower the MTV_40%_, MTV_0.5_, GNTLG_40%_, and GNTLG_0.5_ of the primary tumor. In the group IIIC1-T2B or IIIC2-T2B with lymph node metastasis, although T staging was lower than that in the group IIIB-T3B without lymph node metastasis, the metabolic parameters of the local tumor were comparable. Even using two different measurement methods, we still got similar results. In other words, the difference between the group IIIC and the other groups was only the lymph node metastasis. Our study showed that the staging criteria for stage IIIC disease (Stage IIIC disease is directly related to pelvic and para-aortic metastatic lymph nodes regardless of the T stage) seemed to be more reasonable.

To our knowledge, this is the first study to evaluate the differences in the ^18^F-FDGPET/CT metabolic parameters of primary tumors since the new staging system was revised. Our study was retrospective, FIGO staging was performed by clinicians according to imaging examinations while the invasive range of the primary tumor and lymph node metastasis were not confirmed by pathology, which could lead to inaccurate staging. This aspect is particularly important because histologic analysis generally shows higher sensitivity for detecting nodal metastasis than radiologic studies (32). Therefore, we did not test the correlation between metabolic parameters of the primary tumors and lymph node metastasis due to lacking of pathology as a gold standard. In addition, we had a shorter follow-up period, so we did not perform outcome analysis. The stage III was incomplete (Our study lacked samples for IIIA-T3a, IIIC1-T1, IIIC1-T3a, IIIC2-T1 and IIIC2-T3a, etc.). We only compared the differences between the above five groups, further follow-up studies with larger sample numbers are therefore warranted.

A number of limitations of this study should be acknowledged. Firstly, as mentioned above, it is difficult to validate any method of tumor quantification against the published MTV and TLG due to the lack of a true gold standard. Secondly, the retrospective nature of the analysis led to inconsistencies in uptake time and the injected ^18^F-FDG dose varied over time. Thirdly, Moreover, outcome analysis was not performed. While we investigated differences in the ^18^F-FDG PET/CT metabolic parameters of primary tumors among five groups with stage III disease (IIIB-T3B, IIIC1-T2B, IIIC1-T3B, IIIC2-T2B, IIIC2-T3B), the patient population of stage IIIA was too small and heterogeneous (in terms of stage and histology) to allow meaningful assessment of potential correlation with progression-free and overall survival. To address this issue, follow-up studies on larger homogeneous patient cohorts are planned.

## Conclusion

In this study, we compared the ^18^F-FDG-PET/CT metabolic parameters measured based on fixed percentage threshold of SUVmax and AT-AIA in patients with LACC. Our data showed that MTV measured based on fixed percentage threshold was smaller than that based on AT-AIA. On the other hand, the TLG and GNTLG were not significantly different between the two groups, the two measurement methods had no effect on TLG and GNTLG, which differs from the previous studies. MTV assessment using various methods has been reported. Validation of methods for tumor quantification against the established MTV and TLG parameters is a significant challenge due to the lack of a true gold standard, and selection of the appropriate measurement method to obtain ^18^F-FDG-PET/CT metabolic parameters is important.

Our results showed that when the stage III patients have the same T stage, their metabolic parameters of local tumor were not significantly different, regardless of the presence or absence of lymph node metastasis, location of metastatic lymph nodes in the pelvic cavity or para-abdominal aorta. In stage IIIC1 or stage IIIC2, all patients with lymph node metastasis, the lower the T stage, the lower the MTV_40%_, MTV_0.5_, GNTLG_40%_, and GNTLG_0.5_ of the primary tumor. In the group IIIC1-T2B or IIIC2-T2B with lymph node metastasis, although T staging was lower than that in the group IIIB-T3B without lymph node metastasis, the metabolic parameters of the local tumor were comparable. Staging according to the revised FIGO staging system, stage III patients with lymph node metastasis did not have higher ^18^F-FDG uptake than those without lymph node metastasis when they had the same T stage. Even if the FIGO stage was the same in stage IIIC1 or IIIC2, there were significant differences in some metabolic parameters if the T stage is different. Although patients in the group IIIC1-T2B or IIIC2-T2B with lymph node metastasis had lower T stage than that in the group IIIB-T3B without lymph node metastasis, they did not have lower ^18^F-FDG uptake. Even using two different measurement methods, we still got similar results. In other words, we speculated that the difference between the group IIIC and the other groups was only the lymph node metastasis in our study. The collective results imply that the revised FIGO staging system for stage III cervical cancer (Stage IIIC disease is directly related to pelvic and para-aortic metastatic lymph nodes regardless of the T stage) is more reasonable to an extent. Further research on larger patient cohorts is warranted to validate this conclusion.

## Data Availability Statement

The original contributions presented in the study are included in the article/supplementary material, further inquiries can be directed to the corresponding author/s.

## Ethics Statement

Written informed consent was obtained from each participant. The studies involving human participants were reviewed and approved by Ethical Committee of Jiangsu Cancer Hospital.

## Author Contributions

YZ and YH: study concept and design, analysis, and interpretation of data. YZ and SZ: data collection. YZ, YH, and CC: drafting and critical revision of the manuscript. All authors contributed to the article and approved the submitted version.

## Conflict of Interest

The authors declare that the research was conducted in the absence of any commercial or financial relationships that could be construed as a potential conflict of interest.

## Publisher's Note

All claims expressed in this article are solely those of the authors and do not necessarily represent those of their affiliated organizations, or those of the publisher, the editors and the reviewers. Any product that may be evaluated in this article, or claim that may be made by its manufacturer, is not guaranteed or endorsed by the publisher.
